# Protein expression from unintegrated HIV-1 DNA introduces bias in primary *in vitro* post-integration latency models

**DOI:** 10.1038/srep38329

**Published:** 2016-12-02

**Authors:** Pawel Bonczkowski, Marie-Angélique De Scheerder, Eva Malatinkova, Alexandra Borch, Zora Melkova, Renate Koenig, Ward De Spiegelaere, Linos Vandekerckhove

**Affiliations:** 1 HIV Cure Research Center, Department of Internal Medicine, Faculty of Medicine and Health Sciences, Ghent University and Ghent University Hospital, Ghent, Belgium; 2 Paul-Ehrlich-Institut, Paul-Ehrlich-Strasse 51-59, 63225 Langen, Germany; 3 Department of Immunology and Microbiology, 1st Medical Faculty, Charles University, Studnickova 7, 128 00 Prague 2, Czech Republic; 4 Immunity and Pathogenesis Program, Sanford Burnham Prebys Medical Discovery Institute, La Jolla, CA 92037, USA; 5 Department of Morphology, Faculty of Veterinary Sciences, Ghent University, Merelbeke, Belgium

## Abstract

To understand the persistence of latently HIV-1 infected cells in virally suppressed infected patients, a number of *in vitro* models of HIV latency have been developed. In an attempt to mimic the *in vivo* situation as closely as possible, several models use primary cells and replication-competent viruses in combination with antiretroviral compounds to prevent ongoing replication. Latency is subsequently measured by HIV RNA and/or protein production after cellular activation. To discriminate between pre- and post-integration latency, integrase inhibitors are routinely used, preventing novel integrations upon cellular activation. Here, we show that this choice of antiretrovirals may still cause a bias of pre-integration latency in these models, as unintegrated HIV DNA can form and directly contribute to the levels of HIV RNA and protein production. We further show that the addition of reverse transcriptase inhibitors effectively suppresses the levels of episomal HIV DNA (as measured by 2-LTR circles) and decreases the levels of HIV transcription. Consequently, we show that latency levels described in models that only use integrase inhibitors may be overestimated. The inclusion of additional control conditions, such as 2-LTR quantification and the addition of reverse transcriptase inhibitors, is crucial to fully elucidate the actual levels of post-integration latency.

In HIV-1 infected individuals, combination antiretroviral therapy (ART) is able to suppress HIV replication but it fails to clear the virus. This is due to a reservoir of latently infected cells that evade the host immune response and persist throughout the lifetime of infected patients. HIV-1 latency is a dynamic state defined by infected cells containing integrated provirus that does not produce progeny viral particles, but remains capable to do so^1^. Latent HIV reservoirs are intensively studied as they are considered as the last step on the road to full HIV cure^2^.

It is estimated that for every million resting CD4+ T cells only one latently infected cell can be found in patients on long term ART^3^. This extremely low frequency of cells combined with the lack of methods capable to specifically target or isolate these cells underscores the need for *in vitro* models of HIV latency^4^. HIV latency models are used to study the molecular aspects of HIV latency formation and maintenance, but also to screen for candidate latency reversing agents (LRA) that could reactivate latent HIV in a so-called “shock and kill” strategy to clear HIV reservoirs. Initially, most research was performed on latently infected cell lines with integrated silent proviruses. However, these cell lines may not form a good mimic of the *in vivo* situation. Clonal cells are highly homogeneous and do not reflect the diversity of CD4+ T cell populations *in vivo*^2^. Additional limitations of latent cell lines include the cell cycle status (resting or dividing cells), the inability to change the proliferation status (active or quiescent cells) as well as the clonal HIV integration sites^5^. Recently, a number of latency models have been developed with primary CD4+ T cells and with infectious virus to mimic the *in vivo* situation as closely as possible^6^,^7^,^8^. These models better represent the diversity of CD4 T cells *in vivo* and multiple distinct infections lead to a number of different integrated proviruses in multiple genomic sites. To prevent ongoing replication in primary HIV latency models with infectious virus, ART treatment is routinely performed^5^,^7^,^8^,^9^,^10^,^11^,^12^.

HIV latency can be broadly classified into two forms, i.e. pre- and post-integration latency. Pre-integration latency refers to cells that are infected by HIV but in which the HIV DNA did not yet integrate. This includes viral particles that recently entered the host cell and have not completed reverse transcription, or HIV DNA that has not yet integrated into the genome of the host cell. Post-integration latency refers to cells that harbour integrated HIV DNA that is transcriptionally silent, yet fully competent to produce new viruses upon reactivation. In HIV infected patients, post-integration latency is believed to be the major contributor of the long lived HIV reservoir^1^. However, in cell cultures with primary infected cells, both forms of latency are prevalent.

In addition to pre- and post-integration latency, episomal dead end HIV DNA may also persist as 1- or 2-LTR circles. These circles are formed when reverse transcribed HIV DNA fails to integrate. It was initially assumed that 1- or 2-LTR circles are not stable and eventually disappear^13^, but this hypothesis is now under debate as accumulating data indicate that *in vitro*, this form of episomal HIV DNA appears to be rather stable^14^. Interestingly, RNA transcription from unintegrated HIV DNA circles has been reported^15^,^16^,^17^,^18^,^19^. It has been shown that episomal HIV DNA can lead to the production of HIV proteins and may even generate fully replication competent viruses^20^,^21^,^22^. However, the implications of transcription from 2-LTR circles, especially in the context of HIV latency, have remained underestimated and incompletely understood^19^,^23^.

Integrase inhibitors are commonly used in HIV *in vitro* research. Several latency models include integrase inhibitors treatment to inhibit the step of viral integration before cellular reactivation, assumed to prevent a bias from pre-integration latency. However, this is based on the assumption that unintegrated HIV DNA forms do not contribute to the production of viral transcription and protein production. It is already well known that 2-LTRs accumulate in the presence of INSTI in active infection as well as in treatment intensifications studies *in vivo*^17^,^20^,^21^,^24^,^25^.

The observations of HIV DNA transcription from unintegrated HIV DNA imply that the use of integrase inhibitors may not effectively discern pre- and post-integration latency^18^,^19^. To date, this has not been investigated in any of the latency models. Using a recent model of HIV latency, we here show that latency models may indeed be biased by the contribution of pre-integration latency and this bias may lead to an overestimation of latency levels.

## Results

### Decreased HIV DNA transcription is observed upon activation in the presence of NNRTIs

**Figure 1:**
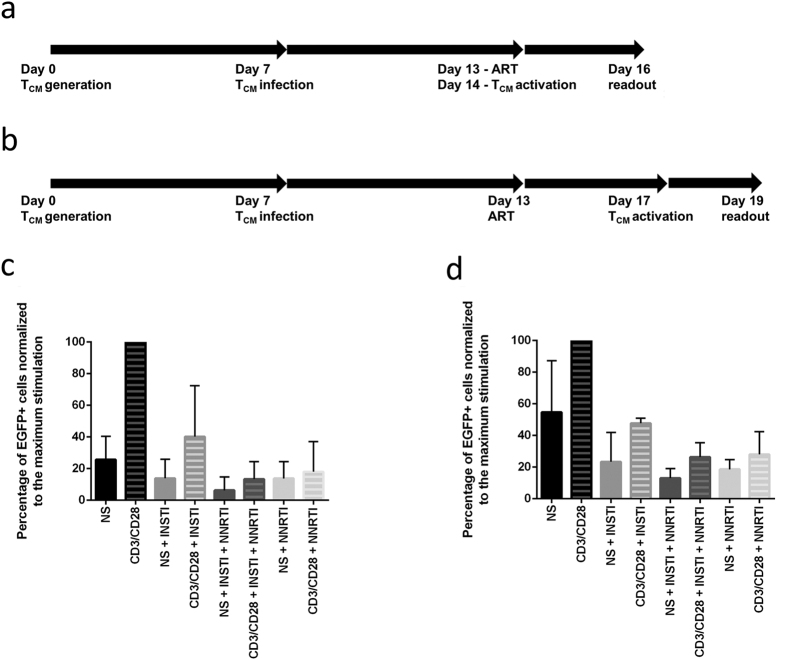
Schematic representation of the modified short (**a**) and original long (**b**) model workflows and corresponding average EGFP expression in the short (**c**) model depicted in 1a and in the long (**d**) model depicted in (**b**). (**a** and **b**) legend: T_CM_–central memory T cells. (**c** and **d**) legend: NS–cells not stimulated (full bars). CD3/CD28–cells activated with αCD3/CD28 microbeads (striped bars), INSTI–integrase strand transfer inhibitors treatment. NNRTI–non-nucleoside reverse transcriptase inhibitor treatment. (**c**) Error bars represent the standard deviation (SD) of 7 replicate experiments performed on cells from 7 independent donors. (**d**) Error bars represent the standard deviation of 3 replicate experiments performed on cells from 3 independent donors. The data was normalized to maximum stimulation by CD3/CD28 per experiment.

We followed two alternative workflows for the generation of HIV latently infected T_CM_ cells, based on the T_CM_ model described earlier^6^. In this study, we used a modified short protocol (Fig. 1a) and the original described long protocol (Fig. 1b)^6^. Statistical analysis of the results obtained from the short model performed on cells from seven independent donors (Supplementary Table S1) indicates that the observed difference in EGFP expression between INSTI-treated non-activated and activated cells is significant (p = 0.029). However, the difference between activated and non-activated cells treated with an NNRTI alone (p = 0.33), or INSTI and an NNRTI (p = 0.23) were not significantly different, indicating that the addition of NNRTI removed the effect observed in the condition with INSTI alone. Among the activated cells, the cells treated with INSTI alone expressed EGFP at a significantly higher level compared to activated cells treated with NNRTI alone (p = 0.006) or the combination of INSTI and an NNRTI (p = 0.019). Of note, NNRTIs efavirenz and nevirapine were both used throughout the study, leading to very similar results.

Although the half-life of 2-LTR circles *in vitro* is an ongoing debate^14^,^22^, the time between ART treatment initiation and readout might have an important impact on the outcomes of the model. To show that the observed effects were not due to our modified short latency model, we followed the original long protocol^6^ in three replicates with independent donor cells. This original model involves a longer period of ART treatment before cellular reactivation (Fig. 1b), possibly decreasing the bias introduced by unstable forms of unintegrated HIV DNA. However, the outcome of this model (Fig. 1d) was similar to the short model as outlined in Fig. 1a and c. It is worth mentioning that the donor cells used for the experiments presented in Fig. 1d were infected at a higher rate than some of the donor cells shown in Fig. 1c. This natural variation between primary cells led to slightly higher EGFP expression across the test conditions in Fig. 1d.

### The presence of distinct EGFP_high_ and EGFP_dim_ populations in activated T cells

**Figure 2:**
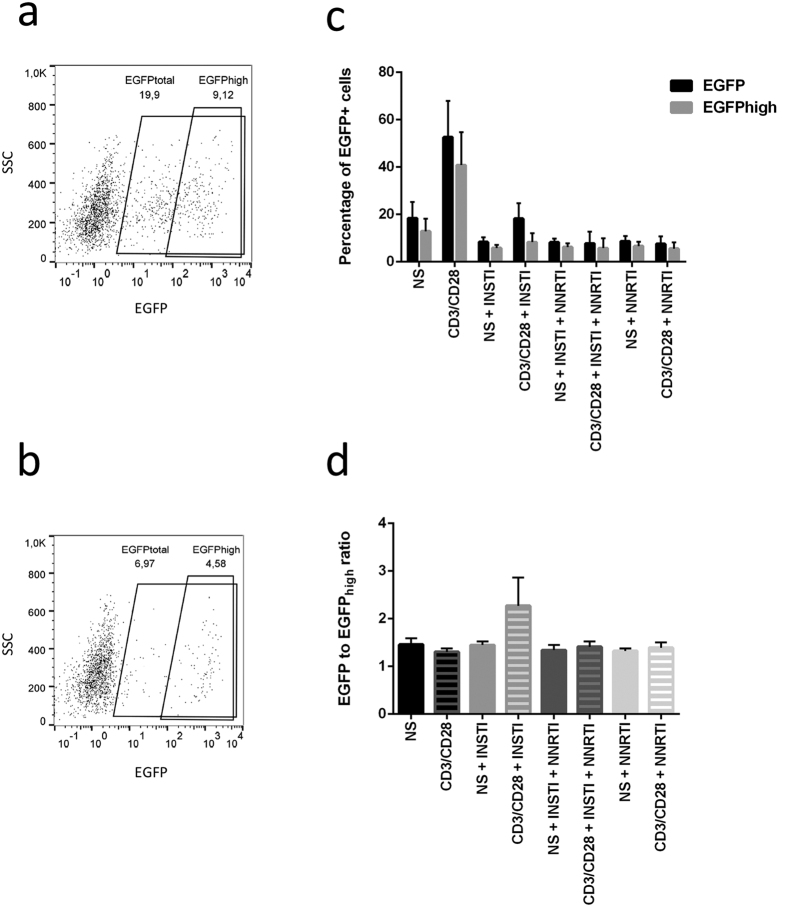
The levels of total EGFP and EGFP_high_ in the short latency model. (**a** and **b**) Flow cytometry readout showing distinct EGFP+ fractions, i.e. the total EGFP (larger gate) and EGFP_high_ (smaller gate). The percentages indicate cells expressing EGFP. (**c** and **d**) legend: NS–cells not stimulated with aCD3/CD28 activator microbeads (full bars). CD3/CD28–cells activated with aCD3/CD28 microbeads (shaded bars). INSTI–integrase strand transfer inhibitors treatment. NNRTI–non-nucleoside reverse transcriptase inhibitor treatment. (**a**) Cells treated with INSTI and activated with anti-CD3/CD28 show high levels of EGFP_dim_. (**b**) Levels of EGFP_dim_ are considerably lower in cells treated with RT inhibitors before activation. (**c**) Levels of total EGFP (black) and EGFP_high_ (grey) across experimental conditions in the model. (**d**) Ratios between EGFP total and EGFP_high_. Error bars represent the SD of 4 replicate experiments performed on cells from 4 independent donors.

The EGFP + cells appeared as 2 distinct populations (Fig. 2a–c, Supplementary Fig. S2) differing by the intensity of the signal (MFI). This phenomenon was previously observed by Trinité *et al*. who showed that unintegrated HIV DNA may cause protein production, but at lower levels compared to integrated HIV DNA^19^. The ratios between total EGFP_total_ and EGFP_high_ were calculated (Fig. 2d). For samples representing most test conditions of the latency model, the ratio approximates 1.5; however, the cells activated in the presence of INSTI show a higher proportion of EGFP_dim_ with the ratios approximating 2. Kruskal-Wallis test performed on these ratios indicates that the differences in EGFP ratios observed are significant (p = 0.020). Interestingly, treatment with reverse-transcriptase inhibitors decreased the levels of EGFP_dim_ in activated cells, indicating that the blockage of reverse transcription suppressed the generation of EGFP_dim_ cells.

### NNRTI treatment largely decreases the levels of 2-LTR circles in activated T cells

**Figure 3:**
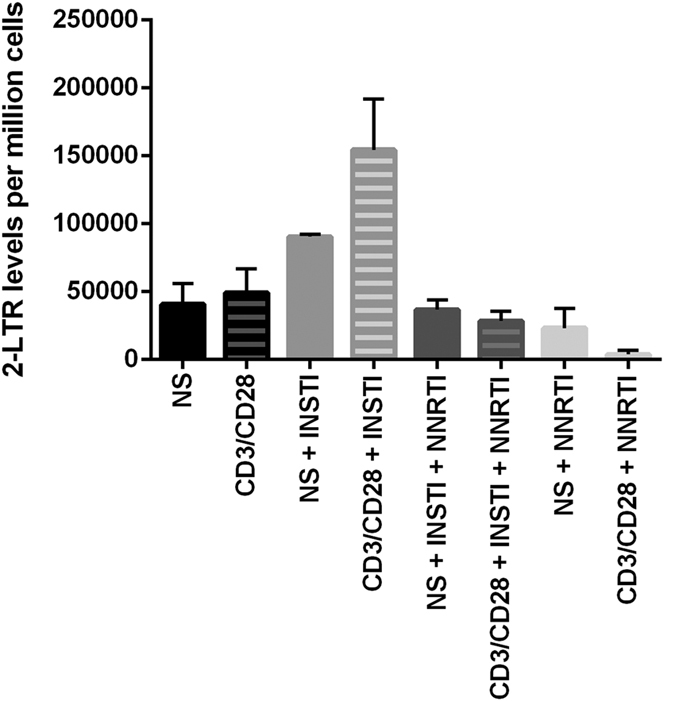
Levels of 2-LTR circles across test conditions as quantified by ddPCR. These levels correspond with the levels of EGFP signal measured by flow cytometry. Error bars represent the standard error of the mean (SEM) of 2 replicate experiments performed on cells from 2 independent donors. Figure legend: NS–cells not stimulated with aCD3/CD28 activator microbeads (full bars). CD3/CD28–cells activated with aCD3/CD28 microbeads (shaded bars). INSTI–integrase strand transfer inhibitors treatment. NNRTI–non-nucleoside reverse transcriptase inhibitor treatment.

To confirm that INSTI treatment induces higher levels of unintegrated HIV DNA, we quantified the levels of episomal 2-LTR circles across the test conditions (Fig. 3). The EGFP expression across the test conditions in the cells from individual donors used for the quantification of 2-LTR circles is represented in Supplementary Fig. S3. The highest levels of 2-LTRs were found in activated cells treated with INSTI, whereas activation in the presence of NNRTIs did not result in high levels of 2-LTRs. This indicates that excess episomal HIV DNA was formed due to the ongoing reverse transcription and the blockage of HIV integration. Interestingly, activation by CD3/CD28 in the presence of NNRTI alone decreased the levels of 2-LTR circles compared to the conditions with INSTI or INSTI and NNRTI, but also compared to the non-activated condition with NNRTI alone (Fig. 3).

### NNRTI treatment does not influence the expression of activation markers or cell viability and has no effect on cellular reactivation

**Figure 4:**
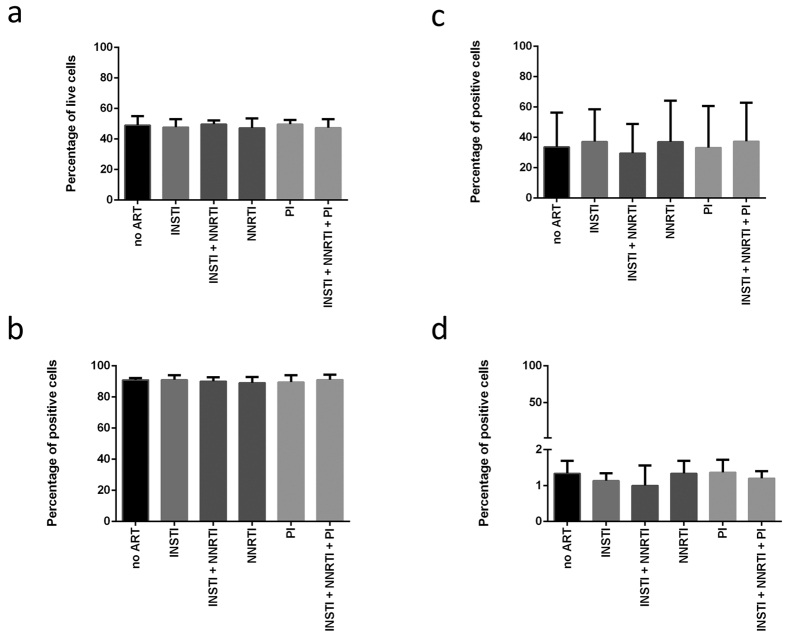
Cell viability and expression of activation markers in the short model. The charts represent cell viability determined by PI staining **(a)** and the expression of activation markers CD38 **(b),** CD25 **(c)** and CD69 **(d)** across test conditions. The experiment was performed on cells from 3 independent donors. Figure legend: no ART–cells not treated with antiretroviral drugs. INSTI–integrase strand transfer inhibitors treatment. NNRTI–non-nucleoside reverse transcriptase inhibitor treatment. PI–protease inhibitor treatment.

To rule out the possibility that NNRTI treatment alone would affect reactivation, we assessed the cell survival and the expression of activation markers CD69, CD25 and CD38 in cells treated with INSTI, NNRTI and PI (protease inhibitor), alone or in combinations (Fig. 4). To obtain data relevant in the context of the latency model, ART was added to the non-infected cells at day 13 of the workflow and the influence of the drugs on cell viability and activation status was assessed 72 h later. The cell survival as determined by negative propidium iodide staining and expression of activation markers throughout experimental conditions were plotted for three independent donors. We observed different levels of activation and cell viability between donors, but within particular donors the differences between experimental conditions were negligible. Additionally, the same treatment was applied to target cells at day 7 of the workflow and its effect was analysed 72 h later. Similarly, high consistency between experimental conditions was observed (data not shown). This indicates that cell treatment with the tested classes of antiretroviral drugs does not influence their activation status or survival.

Finally, to ascertain that ART treatment does not interfere with HIV reactivation, we treated latently infected J-Lat 6.3 cells with individual ARTs as well as their combinations before the reactivation was performed. No difference in the levels of EGFP was observed across test conditions at 24 h and 48 h post-activation (Supplementary Fig. S4), indicating that the ART treatment does not have an influence on the cellular reactivation and HIV transcription.

## Discussion

The present report shows that expression of viral proteins from unintegrated HIV DNA may bias HIV latency models using integrase inhibitors only. Recent studies of HIV infection have shown that unintegrated HIV DNA may indeed produce viral proteins, but its contribution in HIV latency models has not been assessed so far.

*In vitro* models of HIV latency can harbour various forms of pre-integration latency which are either in the process of reverse transcription or still present as viral RNA. Experimental reactivation induces the formation of new HIV DNA which can integrate and start producing new viruses. Integrase inhibitors effectively suppress the integration of these new HIV DNA forms, but cannot prevent the production of viral proteins derived from the episomal HIV DNA forms which accumulate due to the blockage of viral integration. Our data are supported by recent reports that also suggest a contribution of unintegrated DNA to viral gene expression in resting CD4+ T cells^19^,^23^.

Although protease inhibitors and reverse transcriptase inhibitors are used in the course of some of the latency models described to date, most studies only use INSTI treatment upon reactivation to distinguish between already integrated HIV DNA (post-integration latency) and HIV DNA that would integrate as a result of the activation itself (pre-integration latency). Several primary *in vitro* models of HIV latency implemented such treatment^5^,^6^,^7^,^9^,^10^,^12^, however, the possible bias introduced by the inclusion of INSTI has been overlooked. Our results show that treatment of HIV-infected T cells with INSTI before cellular reactivation leads to an increase in EGFP signal normally attributed to reversed latency, but NNRTI acting upstream of EGFP production blocks the formation of this signal. This strongly indicates that it is not, at least in part, the reversed latency that contributes to the increase in EGFP upon activation, but that unintegrated HIV DNA which can be formed in the presence of INSTI and is not formed in the presence of NNRTI is an important contributor to the increase in EGFP protein levels. Interestingly, there is little difference in the levels of EGFP expression upon cellular reactivation between the cells treated with NNRTI alone versus NNRTI and INSTI, indicating that it is the newly formed episomal HIV DNA that is the major source of pre-integration latency and bias in this experimental system.

This observation corresponds with an increased proportion of cells expressing moderate levels of EGFP (EGFPdim) formed in the presence of INSTI, a population that is less abundant in the environment with NNRTI. Additionally, the assessment of 2-LTR levels across the test conditions links the increased ratio of EGFP with higher proportions of episomal, HIV 2-LTR circles in the presence of INSTI, further indicating that the EGFP signal does not originate from the reversed post-integration latency. Conversely, the treatment with NNRTI decreases the levels of unintegrated HIV DNA, consistent with the lower levels of EGFP upon activation in these culture conditions. Similar to what we observed in respect to EGFP levels, activation in the presence of NNRTI leads to a lower number of 2-LTR circles compared to treatment with NNRTI/INSTI or INSTI alone.

In conclusion, treatment with NNRTI acts upstream of INSTI and prevents the formation of any HIV DNA from the viral particles that are still present in the system or from viral RNA that is still in the process of reverse transcription to viral DNA. This indicates that in the condition of INSTI treatment alone, most of the 2-LTRs originate from viral particles or incompletely reverse transcribed viral RNA and not from full pre-integration complexes (PIC) as the latter would not be blocked by NNRTIs and would hence also induce an increase in the levels of 2-LTR circles. Our study shows that the addition of NNRTI shortly before reactivation is crucial to minimize the impact of pre-integration latency in this experimental system and we believe that the combination with INSTI gives the most reliable estimation of post-integration latency levels.

Experiments aimed at excluding the influence of ART on the process of cellular reactivation and downstream EGFP production as well as ascertaining that ART treatment does not influence cellular activation status or survival, confirm the notion that these are not off-target effects of antiretroviral drugs used that lead to the observed phenomena, but rather the desired mode of action of these compounds.

It is also worth mentioning that ART treatment is performed shortly before the reactivation and EGFP readout. If the observed effect of EGFP production from episomal HIV DNA was due to the accumulation of unintegrated HIV DNA in the period between the infection and the ART treatment and activation, there would be no differences between experimental conditions. However, these differences occur with the consistently highest EGFP expression originating from cells treated with INSTI, indicating that it is the increased formation of 2-LTR circles stimulated by CD3/CD28 activation in the presence of integrase inhibitors that leads to the increase in the EGFP levels.

The results of this study are mainly based on EGFP expression from the laboratory strain of HIV containing an *egfp* reading frame upstream of *nef*. This fact may lead to an overestimation of virus-derived EGFP protein since the expression of Nef protein from 2-LTR circles has already been reported earlier^17^,^26^. However, other primary latency models do use the same viral construct harbouring the *egfp* gene in the reading frame of *nef*. Hence, considering the build-up of 2-LTR circles in the presence of INSTI only, we would strongly recommend that these conditions should be very carefully tested and the results validated in other latency models to estimate whether, and to what extent these 2-LTR circles bias the read-out.

At this point, the possible contribution of unintegrated HIV DNA to HIV protein production in HIV latency models has been largely overlooked. Our findings emphasize that other HIV latency models should be validated rigorously by monitoring the levels of 2-LTRs and by evaluating the effect of different classes of ART compounds. We therefore urge researchers performing *in vitro* HIV latency models to include relevant control steps (2-LTR quantification) and conditions (use of NNRTIs) to investigate whether, and to what extent, the contribution of episomal HIV DNA biases the levels of latency in their models.

We would like to emphasize that our findings indicate that the percentage of latently infected cells may be smaller than previously assumed in this and similar latency models. Since these findings may apply for any *in vitro* HIV-1 latency model employing replication-competent strains of HIV and treatment with INSTI, the latency levels described and processed in downstream applications may be largely overestimated. As a result, findings believed to broaden our understanding of HIV latency may originate from a phenomenon other than *in vitro* post-integration HIV latency.

## Methods

### Plasmids and viruses

HIV-1 NLENG1-IRES was a kind gift from Dr. David Levy^18^. The replication-competent virus stocks were prepared by calcium phosphate transfection of 293 T cells (DZSM, Braunschweig, Germany) according to manufacturer’s instructions (Life Technologies).

### Cell isolation and culture

Peripheral blood mononuclear cells (PBMCs) were isolated from the blood of healthy donors following density gradient centrifugation. Donor samples were obtained from Rode Kruis-Vlaanderen, Gent, after informed consent. Blood was diluted in PBS (Lonza, Verviers, Belgium) at 1:1 ratio and 25 ml of diluted blood were slowly added on 12 ml LymphoprepTM (Axis-Shield, Oslo, Norway). This was followed by centrifugation at 770 g for 20 min at RT. Isolated PBMCs were washed twice in PBS and naïve CD4+ T cells were isolated using the Naive CD4+ T Cell Isolation Kit II, human (Miltenyi Biotec, Bergisch Gladbach, Germany) routinely yielding purity exceeding 95% as analysed by flow cytometry (data not shown).

*In vitro* cultured central memory T cells (TCM) were generated from naïve CD4+ T cells cultured for 3 days in non-polarising conditions, i.e. RPMI supplemented with 1 μg/ml anti-IL-4, 2 μg/ml anti-IL-12, 10 ng/ml TGF-β (Peprotech) and anti-CD3/CD28 microbeads (Invitrogen). After three days, magnetic beads were removed with DynaMag™ Spin Magnet (Life Technologies), cells were collected, counted and resuspended in fresh RPMI with 30 IU/ml IL-2 at 1 million cells in 1 ml of RPMI medium before seeding in 96 well plates. Daily medium change was performed for 4 additional days.

Both naïve CD4 T cells and *in vitro* cultured central memory T cells were characterized for the expression of CD45RA and CD45RO (Supplementary Fig. S5). The expression of CD45RA and CD45RO was determined by staining with respective antibodies (Miltenyi Biotech). 1 × 10^5^ cells were transferred to a V-bottom 96 well plate and centrifuged for 5 min, 380 g, at room temperature to remove the medium. Next, anti-CD45RO-APC or anti-CD45RA-VioBlue antibody was added to the cells at a concentration of 10 μl/1 ml PBS, mixed and incubated for 30 min at 4 °C. After 30 min of staining, the unbound antibody was washed away and the cells were analysed by flow cytometry using MACSQuant.

The central memory T cells were infected at day 7 post-isolation by spinoculation in 96-well flat bottom plates. The cells were distributed at 2 × 10^5^ cells/well and viral supernatants were added at 2 × 10^5^ cells/well as quantified by the RT assay. After spinoculation for 90 min, 710 g, 32 °C, the supernatant containing HIV was replaced with fresh RPMI. Cells were transferred to U-bottom 96-well plates for further culture.

J-Lat 6.3 cells (Cat. No 9846, obtained through the NIH AIDS Reagent Program, Division of AIDS, NIAID, NIH) were cultured in IMDM medium (Invitrogen) at a density of 5 × 10^5^–106 cells per 1 ml medium^27^. Medium change was performed twice a week to maintain the desired density of cells. For the experiment investigating the influence of ART treatment on latency reversal, 10^5^ cells were plated in a 96-well plate, treated with the single ART or combinations of drugs and treated with 16 nM PMA for 24 h or 48 h. This was followed by flow cytometric readout.

### General latency model workflow

The study followed two alternative workflows to generate latently infected cells.

Briefly, the modified short workflow involves the isolation of naïve CD4+ T cells at day 0, culturing in non-polarising conditions for 72 h, daily medium change for additional 4 days and viral infection at day 7. The cells are treated with antiretroviral drugs at day 13, and activated at day 14. The flow cytometric readout follows at day 16^12^ (Fig. 1a).

The original long workflow involves the isolation of naïve CD4+ T cells at day 0, culturing in non-polarising conditions for 72 h, daily medium change for additional 4 days and viral infection at day 7 followed by a longer period of antiretroviral treatment, from day 13, cellular activation at day 17 and the readout at day 19^6^ (Fig. 1b).

### Antiretroviral treatment and activation

1 day before activation, cells were distributed at 2 × 10^5^ cells per well in 200 μl medium. To differentiate between pre- and post-integration latency, a fraction of the wells was treated with INSTI (integrase strand transfer inhibitor, raltegravir) at 1 μM (Cat. No 11680 from Merck & Company, Inc.), or 2 μM NNRTI (non-nucleoside reverse-transcriptase inhibitor, efavirenz [Cat. No 4624] or nevirapine [Cat. No 4666] obtained through the NIH AIDS Reagent Program, Division of AIDS, NIAID, NIH). For the assessment of the influence of ART on cell toxicity and activation status, cells were treated with PI (protease inhibitor, ritonavir) at 1 μM (Cat. No 4622). Cells were then stimulated with anti-CD3/CD28 microbeads (Dynabeads^®^ Human T-Activator CD3/CD28, Invitrogen) for 48 h before the readout by flow cytometry.

### Quantification of infected cells and activation markers

The amount of HIV productive cells was quantified by the EGFP reporter protein produced by the HIV lab strain. Briefly, 1 × 10^5^ cells were transferred to a V-bottom 96 well plate and centrifuged for 5 min, 380 g, at room temperature to remove the medium. Next, 100 μl of PBS was added and the cells were analysed by flow cytometry using MACSQuant (Miltenyi Biotech).

The expression of activation markers was determined by staining of CD25, CD69 and CD38 with respective antibodies (BD Biosciences). 1 × 10^5^ cells were transferred to a V-bottom 96 well plate and centrifuged for 5 min, 380 g, at room temperature to remove the medium. Next, 1 μl of anti-CD25-PE, anti-CD38-PE or anti-CD69-FITC (BD Biosciences) antibody in 100 μl of PBS was added to the cells, mixed and incubated for 30 min at 4 °C. After 30 min of staining, the unbound antibody was washed away and the cells were analysed by flow cytometry using MACSQuant.

Flow cytometric readout was performed to identify the levels of expression of activation markers CD25, CD38, CD69 or EGFP. The cells were routinely stained with propidium iodide to exclude dead cells. By gating out all propidium iodide positive cells, only the live cells were included in the analysis. The results of a longitudinal analysis of the expression of activation markers CD25, CD38 and CD69 are presented in Supplementary Fig. S6.

### 2-LTR level quantification

Episomal HIV-1 2-LTR circles were quantified from genomic DNA isolated from TCM using ddPCR as previously described^28^. Episomal DNA was isolated using the QIAprep Spin Miniprep kit (Qiagen). The measurement was performed in duplicate reactions using ddPCR with the QX100™ Droplet Digital™ PCR platform (Bio-Rad, Hercules, California). The ddPCR reaction mix consisted of 2 μl of genomic DNA, 10 μl of 2x ddPCR™ Supermix for probes (Bio-Rad), 500 nM primers (forward primer -5′-CTAACTAGGGAACCCACTGCT-3, reverse primer -5′-GTAGTTCTGCCAATCAGGGAAG-3′) and 250 nM probe (5′-/56-FAM/AGC CTC AAT/ZEN/AAA GCT TGC CTT GAG TGC/3IABkFQ/-3′) in 20 μl final volume. The cycling conditions were as follows: initial denaturation at 95 °C for 5 min, 40 cycles of 95 °C for 30 sec denaturation and elongation 60 sec with a ramp rate of 2.5 °C/sec. Droplets were read by the QX200™ droplet reader (Bio-Rad) and the data was analyzed using a data driven method for threshold determination with the ddpcRquant software in R as previously performed^29^.

### Statistical analysis

Assessment of the statistical significance of the differences in EGFP levels between test conditions was performed using paired t-test (package ‘stats’, version: 3.0.2) and Kruskal–Wallis one-way analysis of variance (package ‘stats’, version: 3.0.2) with RStudio (version 0.99.902).

## Additional Information

**How to cite this article**: Bonczkowski, P. *et al*. Protein expression from unintegrated HIV-1 DNA introduces bias in primary *in vitro* post-integration latency models. *Sci. Rep.* **6**, 38329; doi: 10.1038/srep38329 (2016).

**Publisher’s note:** Springer Nature remains neutral with regard to jurisdictional claims in published maps and institutional affiliations.

## Supplementary Material

Supplementary Information
