# Supplementary material for: Protein expression from unintegrated HIV-1 DNA introduces bias in primary in vitro post-integration latency models
Source: Sci Rep. 2016 Dec 2;6:38329. doi: 10.1038/srep38329 (PMC5133580; doi:10.1038/srep38329)
Supplement: Supplementary Information [file srep38329-s1.pdf]

Protein expression from unintegrated HIV-1 DNA introduces bias in primary in vitro post-integration latency models

Pawel Bonczkowski[1], Marie-Angélique De Scheerder[1], Eva Malatinkova[1], Alexandra Borch[2], Zora Melkova[2,3], Renate Koenig [2,4], Ward De Spiegelaere[1,5], Linos Vandekerckhove[1*]

1. HIV Cure Research Center, Department of Internal Medicine, Faculty of Medicine and Health Sciences, Ghent University and Ghent University Hospital, Ghent, Belgium.

2. Paul-Ehrlich-Institut, Paul-Ehrlich-Strasse 51-59, 63225 Langen, Germany

3. Department of Immunology and Microbiology, 1st Medical Faculty, Charles University, Studnickova 7, 128 00 Prague 2, Czech Republic

4. Immunity and Pathogenesis Program, Sanford Burnham Prebys Medical Discovery Institute, La Jolla, CA 92037, USA

5. Department of Morphology, Faculty of Veterinary Sciences, Ghent University, Merelbeke, Belgium

* - corresponding author

NS + INSTI	NS + INSTI + NNRTI	NS + NNRTI	CD3/CD28	CD3/CD28 + INSTI	CD3/CD28 + INSTI + NNRTI	CD3/CD28 + NNRTI	
0.00226	0.00667	0.00059	1.04E-05	0.1142	0.0203	0.09441	**NS**
	0.1504	0.9666	1.35E-06	0.02851	0.2346	0.3934	**NS + INSTI**
		0.1112	1.07E-07	0.03172	0.2346	0.187	**NS + INSTI + NNRTI**
			6.42E-07	0.02117	0.8629	0.3252	**NS + NNRTI**
				0.00268	7.88E-07	2.76E-05	**CD3/CD28**
					0.01865	0.00593	**CD3/CD28 + INSTI**
						0.247	**CD3/CD28 + INSTI + NNRTI**

Table S1. The results of the statistical analysis performed on the EGFP expression levels in the short model. The numbers represent the p-values of particular analyses performed with Wilcoxon signed-rank test.

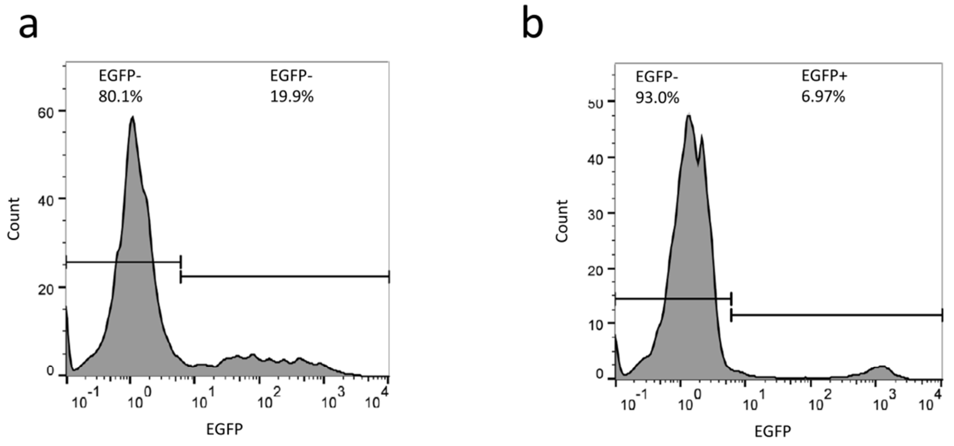

Figure S2. Histogram plots representing the data shown in Fig. 2a and 2b. Refer to Fig. 2 for description.

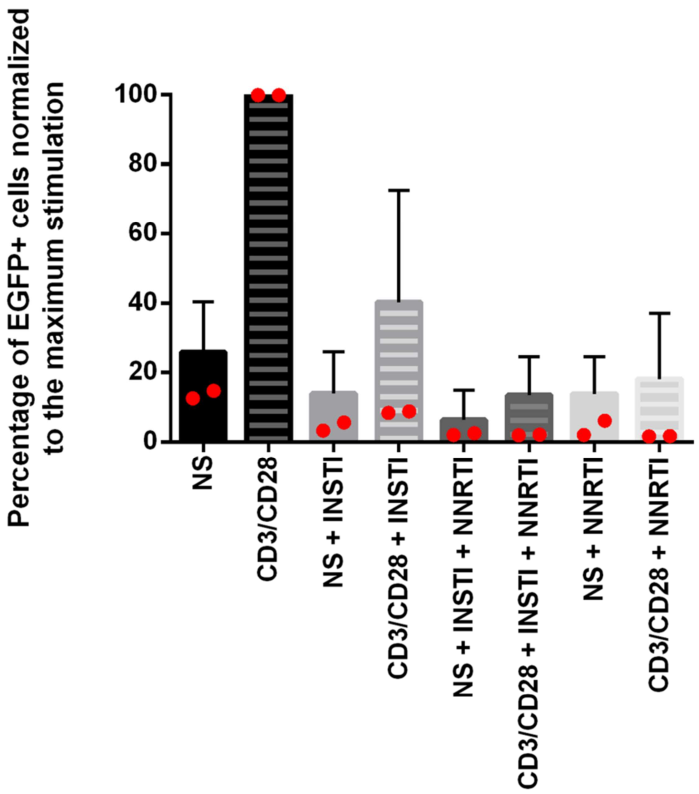

Figure S3. Average EGFP expression in the short latency model. The red events mark the EGFP levels in the cells used for the 2-LTR levels assessment. Figure legend: NS – cells not stimulated with aCD3/CD28 activator microbeads (full bars). CD3/CD28 – cells activated with aCD3/CD28 microbeads (shaded bars). INSTI – integrase strand transfer inhibitors treatment. NNRTI – non-nucleoside reverse transcriptase inhibitor treatment. Error bars represent the standard deviation (SD) of 7 replicate experiments performed on cells from 7 independent donors.

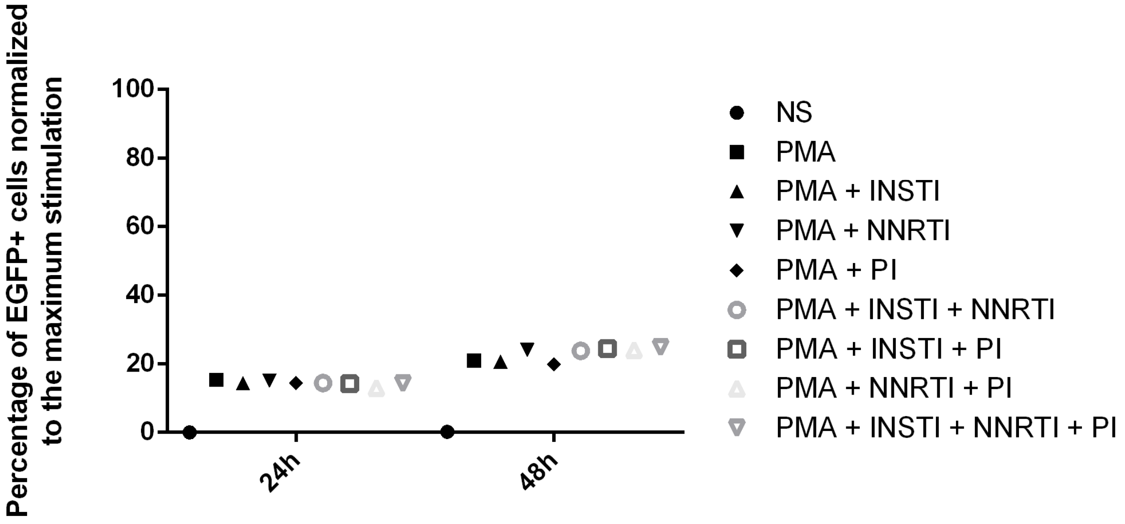

Figure S4. The expression of EGFP in J-Lat 6.3 cells treated with individual ARTs and their combinations before reactivation

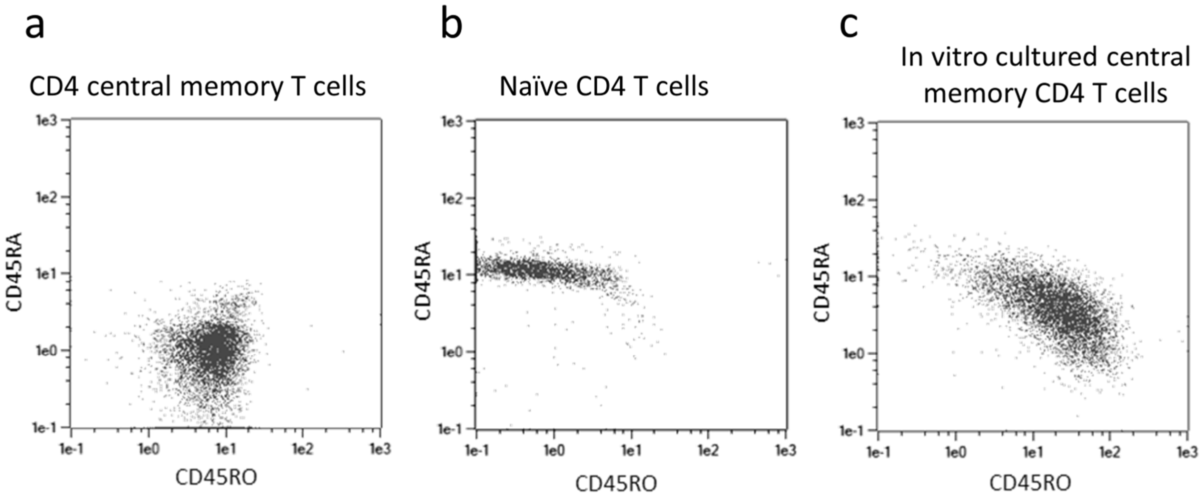

Figure S5. Expression of CD45RA and CD45RO in central memory (a), naïve (b) and non-polarised (c) CD4 T cells. Routine characterization of freshly isolated naïve CD4 T cells shows high expression of CD45RA and low levels of CD45RO. Upon *in vitro* differentiation of these cells to non-polarised CD4 T cells, the phenotype changes and the cells express high levels of both CD45RO characteristic for *in vivo* central memory T cells and CD45RA.

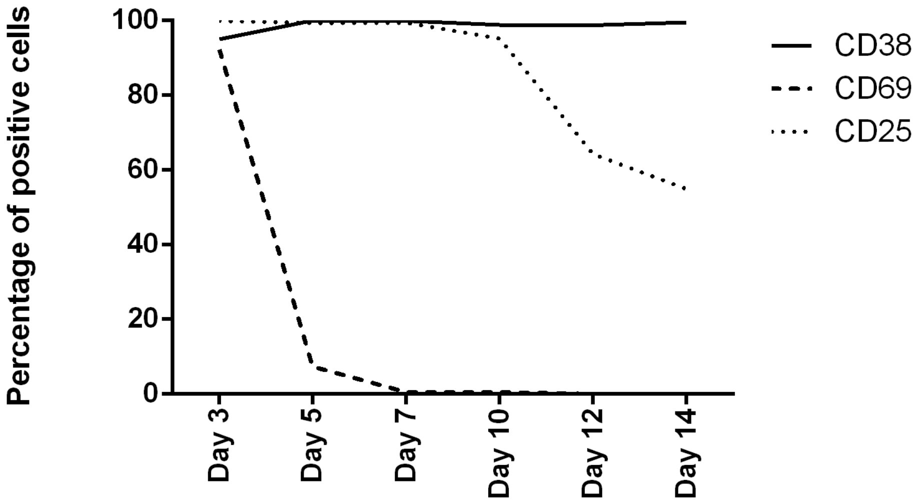

Figure S6. Expression of activation markers in the course of the short model. Day 3 represents the time when T cells with a central memory phenotype are generated and the first point when activation markers can be measure. Activation marker CD38 is expressed at high levels throughout the model, the expression of CD25 routinely declines around day 10, while the activation marker CD69 is characterized by an early decline leading to no expression at day 7 of the workflow.
